# Evaluation of N-terminal Pro-B-Type Natriuretic Peptide (NT-proBNP) as a Biomarker of Cardiac Dysfunction Across Heart Failure Phenotypes

**DOI:** 10.7759/cureus.96447

**Published:** 2025-11-09

**Authors:** Ambreen Fatima, Makhdoom Haris Mansoor, Ahsan Munir, Bilawal Ali, Hamna Maryam, Muhammad Salman Tariq

**Affiliations:** 1 Cardiology, Shalamar Hospital, Lahore, PAK; 2 Internal Medicine, Pakistan Kidney and Liver Institute and Research Center, Lahore, PAK; 3 Biochemistry, Wah Medical College, Wah, PAK; 4 Internal Medicine, DHQ Teaching Hospital, Dera Ghazi Khan, PAK; 5 Internal Medicine, Shalamar Hospital, Lahore, PAK; 6 Cardiology, Jinnah Hospital, Lahore, Lahore, PAK

**Keywords:** atrial fibrillation, heart failure, ischemic heart disease, left ventricular ejection fraction, nt-probnp

## Abstract

Background

Heart failure (HF) is a global health problem with high morbidity and mortality. N-terminal pro-B-type natriuretic peptide (NT-proBNP) is a secondary data point to left ventricular ejection fraction (LVEF) that captures all the aspects of cardiac dysfunction, which is not included in systolic performance. There is significant clinical value in using NT-proBNP and LVEF together.

Objective

To ascertain the association between NT-proBNP levels and LVEF in patients with HF and to identify clinical predictors of elevated NT-proBNP concentrations.

Methods

This retrospective observational study was conducted at Shalamar Hospital, Lahore, Pakistan, from February 2023 to February 2025. This research involved 165 participants who had been diagnosed with HF. Electronic medical records provided demographic, clinical, lab, and echocardiographic data. The patients were grouped into HF with reduced ejection fraction (HFrEF, <40%), mildly reduced ejection fraction (HFmrEF, 41-49%), or preserved ejection fraction (HFpEF, ≥50%).

Results

Among the 165 patients, 82 (49.7) patients were HFrEF, 37 (22.4) patients were HFmrEF, and 46 (27.9) patients were HFpEF. HFrEF (4,020 pg/mL (interquartile range (IQR): 2,210-6,580)) had the highest median NT-proBNP concentration when compared to HFmrEF (2,350 pg/mL (IQR: 1,180-3,640)) and HFpEF (1,780 pg/mL (IQR: 910-2,950)) (p < 0.001). NT-proBNP had a significant negative relationship with LVEF in the general cohort (r = -0.52, p<0.001), with the highest value of r -0.61, and p -0.001 in HFrEF.

Conclusion

In HF, NT-proBNP serves as a valuable biomarker that complements echocardiographic assessment by capturing global cardiac stress beyond systolic function alone. In our cohort, NT-proBNP levels demonstrated a significant association with reduced LVEF and correlated with key clinical predictors, reinforcing its role as an accessible and reliable tool for comprehensive cardiac evaluation. Its integration into routine practice may enhance diagnostic accuracy, guide risk stratification, and support individualized treatment planning within our population context.

## Introduction

Heart failure (HF) is a major global health concern, with an estimated 64 million individuals affected worldwide, many of whom experience recurrent hospitalizations, impaired quality of life, and premature mortality despite advances in healthcare [1]. This burden continues to grow due to an aging population, longer survival after acute coronary syndromes, and increasing prevalence of cardiovascular risk factors such as hypertension, diabetes, and obesity [2]. Despite therapeutic advancements, HF remains associated with poor prognosis, and its five-year mortality rate is comparable to that of many malignancies. Consequently, there is a strong need to identify reliable diagnostic tools and prognostic markers that can optimize early management and improve outcomes [3].

The pathophysiology of HF is multifactorial, encompassing structural, functional, and neurohormonal alterations. Activation of compensatory mechanisms, particularly the renin-angiotensin-aldosterone system (RAAS) and sympathetic nervous system, initially maintains cardiac output but ultimately results in maladaptive remodeling, increased wall stress, and myocardial dysfunction [4]. These processes stimulate the release of natriuretic peptides - brain natriuretic peptide (BNP) and its inactive fragment N-terminal proBNP (NT-proBNP) - which serve as biochemical indicators of ventricular strain and congestion. NT-proBNP is biologically inactive, has a longer plasma half-life, and exhibits greater stability than BNP, making it a more reliable biomarker for clinical assessment [5]. Elevated NT-proBNP levels strongly correlate with HF diagnosis, severity, and prognosis, and are included in international HF guidelines as an adjunct to clinical and imaging evaluation [6].

In addition to biochemical assessment, left ventricular ejection fraction (LVEF) remains the primary imaging measure of systolic function and a cornerstone in HF diagnosis and classification. Current consensus divides HF into three groups: HF with reduced ejection fraction (HFrEF), HF with mildly reduced ejection fraction (HFmrEF, 41-49%), and HF with preserved ejection fraction (HFpEF) [7]. This classification has therapeutic implications, as most evidence-based pharmacologic therapies, such as beta-blockers, angiotensin-converting-enzyme (ACE) inhibitors, and mineralocorticoid receptor antagonists, are effective mainly in HFrEF [8]. Conversely, HFpEF remains challenging to manage, reflecting the syndrome’s heterogeneity and emphasizing the need for additional indicators beyond LVEF alone [9].

Previous studies have demonstrated an overall inverse relationship between NT-proBNP levels and LVEF, where elevated NT-proBNP corresponds to reduced contractile function [10]. However, this relationship is not linear and may be influenced by multiple clinical variables, including diastolic dysfunction, atrial fibrillation, renal impairment, and ventricular stiffness [11]. These findings indicate that NT-proBNP provides complementary information to LVEF, reflecting global cardiac stress and dysfunction not captured by systolic performance alone [12,13].

Importantly, emerging evidence highlights that social determinants, such as socioeconomic status, health literacy, and access to care, significantly influence HF outcomes, contributing to disparities in hospitalization rates and long-term prognosis [14]. Incorporating these factors into HF assessment frameworks is increasingly recognized as essential for developing equitable, patient-centered management strategies.

Objective

To ascertain the association between NT-proBNP levels and LVEF in patients with HF and to identify clinical predictors of elevated NT-proBNP concentrations.

## Materials and methods

Methodology

This was a retrospective observational study conducted at Shalamar Hospital, Lahore, Pakistan, from February 2023 to February 2025. A total of 165 patients with a confirmed clinical diagnosis of HF were included.

Inclusion and exclusion criteria

Patients aged 18 years or older with a diagnosis of HF, an available NT-proBNP measurement, and documented echocardiographic assessment of LVEF during the study period were included. Patients were excluded if they had incomplete records, missing NT-proBNP or LVEF data, advanced chronic kidney disease (stage IV-V), acute coronary syndrome at the time of sampling, or other acute conditions known to independently elevate NT-proBNP, such as pulmonary embolism or sepsis, in order to minimize confounding.

Data collection

Data were extracted from the hospital’s electronic records using a standardized collection form. Echocardiographic measurements, laboratory values (NT-proBNP, serum creatinine), clinical characteristics (hypertension, diabetes mellitus, atrial fibrillation, ischemic heart disease), and demographic information (age, sex, and residence) were gathered. NT-proBNP levels were recorded from blood samples taken at admission or outpatient visits. Echocardiographic assessment was performed manually by two experienced cardiologists using standardized American Society of Echocardiography (ASE) protocols, and LVEF was measured by the biplane Simpson’s method, with an inter-observer variation of less than 5%. Patients in this study were stratified into three groups based on their LVEF. Those with a lower LVEF of less than 40% were considered to have a reduced ejection fraction (HFrEF). With LVEFs ranging from 41% to 49%, mildly reduced ejection fraction (HFmrEF) patients were identified. Finally, individuals with preserved ejection fraction (HFpEF) were defined as having an LVEF of 50% or greater. The primary outcome was to ascertain the relationship between NT-proBNP levels and LVEF across different HF categories. Secondary outcomes included quantifying the strength of this correlation and performing subgroup analyses based on relevant clinical variables.

Statistical analysis

The Statistical Package for the Social Sciences (SPSS) version 27.0 (IBM Corp., Armonk, NY, USA) was used for all analyses. Continuous variables were tested for normality using the Shapiro-Wilk test. Normally distributed continuous variables were presented as mean ± standard deviation (SD), while skewed data were expressed as median with interquartile range (IQR). Categorical variables were summarized as frequencies and percentages.

Between-group comparisons of categorical variables were performed using the Chi-square (χ^2^) test, with results reported as χ^2^ statistic and p-value. Continuous variables were compared using the independent-samples t-test (reported as t statistic and p-value) when two groups were involved, or one-way analysis of variance (ANOVA) when more than two groups were compared (reported as F statistic and p-value). Correlations between continuous variables were assessed using Pearson’s correlation coefficient (r) with corresponding p-values.

Multivariable predictors of elevated NT-proBNP were evaluated using binary logistic regression. Adjusted odds ratios (AORs) with 95% confidence intervals (CIs), Wald χ^2^ statistics, and p-values were reported for each covariate. A p-value <0.05 was considered statistically significant.

## Results

A total of 165 patients with HF were included in the analysis. The mean age was 61.3 ± 12.4 years, and 98 (59.4%) were male. When age was categorized, 21 patients (12.7%) were aged 18-40 years, 38 (23.0%) were 41-55 years, and 106 (64.2%) were older than 55 years. The distribution suggests that the majority of HF cases occurred in older adults, consistent with the typical epidemiological profile of HF. Hypertension was present in 102 patients (61.8%), diabetes mellitus in 74 (44.8%), ischemic heart disease in 89 (53.9%), and atrial fibrillation in 34 (20.6%). The median serum creatinine level was 1.2 mg/dL (IQR: 0.9-1.7). Hypertension was the most common comorbidity, present in 102 patients (61.8%), followed by ischemic heart disease in 89 patients (53.9%) and diabetes mellitus in 74 patients (44.8%). Atrial fibrillation was identified in 34 patients (20.6%) (Table 1).

**Table 1:** Baseline Demographic and Clinical Characteristics of Patients (N=165) Independent t-tests were used for normally distributed continuous variables, chi-square tests for categorical variables, and Mann-Whitney U tests for non-parametric continuous data.

Variable	Total (N=165)	Male (n=98)	Female (n=67)	Test Statistic	P-value
Age, years (Mean ± SD)	61.3 ± 12.4	62.1 ± 11.8	60.1 ± 13.2	t = 0.98	0.33
Hypertension, n (%)	102 (61.8)	62 (63.3)	40 (59.7)	χ² = 0.20	0.65
Diabetes mellitus, n (%)	74 (44.8)	44 (44.9)	30 (44.8)	χ² = 0.00	0.99
Ischemic heart disease, n (%)	89 (53.9)	58 (59.2)	31 (46.3)	χ² = 2.39	0.12
Atrial fibrillation, n (%)	34 (20.6)	21 (21.4)	13 (19.4)	χ² = 0.08	0.78
Serum creatinine, mg/dL (median, IQR)	1.2 (0.9-1.7)	1.3 (1.0-1.8)	1.1 (0.8-1.5)	Mann-Whitney U = 2911.0	0.09

When stratified by LVEF, nearly half of the patients (49.7%) had HFrEF, 22.4% had HFmrEF, and 27.9% had HFpEF. The mean LVEF in the entire population was 41.6 ± 12.9%. NT-proBNP levels differed significantly across groups, with the highest concentrations in HFrEF (median 4,020 pg/mL, IQR: 2,210-6,580), followed by HFmrEF (2,350 pg/mL, IQR: 1,180-3,640), and HFpEF (1,780 pg/mL, IQR: 910-2,950) (p < 0.001) (Table 2).

**Table 2:** Distribution of Left Ventricular Ejection Fraction (LVEF) and NT-proBNP Levels One-way ANOVA was applied for LVEF, and the Kruskal-Wallis test was applied for NT-proBNP. ANOVA: analysis of variance; NT-proBNP: N-terminal pro-B-type natriuretic peptide; HFrEF: heart failure with reduced ejection fraction; HFmrEF: heart failure with mildly reduced ejection fraction; HFpEF: heart failure with preserved ejection fraction

LVEF Category	n (%)	LVEF % (Mean ± SD)	NT-proBNP pg/mL (Median, IQR)	Test Statistic	P-value
HFrEF (<40%)	82 (49.7)	32.4 ± 5.1	4,020 (2,210-6,580)	F = 512.4	<0.001
HFmrEF (41-49%)	37 (22.4)	44.6 ± 2.3	2,350 (1,180-3,640)	-	-
HFpEF (≥50%)	46 (27.9)	55.2 ± 3.8	1,780 (910-2,950)	-	-
Total	165 (100)	41.6 ± 12.9	2,640 (1,210-4,890)	-	-

The correlation between NT-proBNP and LVEF remained strong and statistically significant in the reduced EF group (r = -0.57, p < 0.001), while no significant association was observed in the preserved EF group (r = -0.19, p = 0.17) (Table 3).

**Table 3:** Correlation Between NT-proBNP Levels and LVEF Across Heart Failure Categories Pearson correlation coefficients with corresponding t-statistics. NT-proBNP: N-terminal pro-B-type natriuretic peptide; HFrEF: heart failure with reduced ejection fraction; HFmrEF: heart failure with mildly reduced ejection fraction; HFpEF: heart failure with preserved ejection fraction; LVEF: left ventricular ejection fraction

Category	Correlation Coefficient (r)	Test Statistic	P-value
Overall cohort	-0.52	t = -8.24	<0.001
Reduced EF (HFrEF + HFmrEF)	-0.57	t = -7.83	<0.001
Preserved EF (HFpEF)	-0.19	t = -1.40	0.17

Patients with HFrEF were more likely to be male (68.3%) compared with HFpEF (41.3%) (p = 0.01). Ischemic heart disease was most prevalent in the HFrEF group (70.7%) compared with 48.6% in HFmrEF and 28.3% in HFpEF (p < 0.001) (Table 4).

**Table 4:** Clinical Characteristics by LVEF Category One-way ANOVA for continuous variables and Chi-square for categorical variables. ANOVA: analysis of variance; HFrEF: heart failure with reduced ejection fraction; HFmrEF: heart failure with mildly reduced ejection fraction; HFpEF: heart failure with preserved ejection fraction; LVEF: left ventricular ejection fraction

Variable	HFrEF (n=82)	HFmrEF (n=37)	HFpEF (n=46)	Test Statistic	P-value
Age, years (Mean ± SD)	63.1 ± 11.7	60.2 ± 12.3	59.8 ± 13.1	F = 2.15	0.12
Male sex, n (%)	56 (68.3)	23 (62.2)	19 (41.3)	χ² = 9.34	0.01
Hypertension, n (%)	45 (54.9)	25 (67.6)	32 (69.6)	χ² = 3.37	0.18
Diabetes mellitus, n (%)	36 (43.9)	17 (45.9)	21 (45.7)	χ² = 0.12	0.94
Ischemic heart disease, n (%)	58 (70.7)	18 (48.6)	13 (28.3)	χ² = 21.6	<0.001
Atrial fibrillation, n (%)	19 (23.2)	7 (18.9)	8 (17.4)	χ² = 0.68	0.71

In the multivariable logistic regression model, several predictors of elevated NT-proBNP (>3,000 pg/mL) were identified. Age was associated with higher NT-proBNP levels, with each 10-year increase conferring a 1.28-fold higher risk (95% CI: 1.05-1.64, p = 0.02). Ischemic heart disease (AOR: 2.11, 95% CI: 1.18-3.79, p = 0.01), atrial fibrillation (AOR: 2.65, 95% CI: 1.22-5.73, p = 0.01), reduced EF (HFrEF; AOR: 3.42, 95% CI: 1.82-6.46, p < 0.001), and renal dysfunction (AOR: 2.88, 95% CI: 1.59-5.23, p < 0.001) independently predicted markedly elevated NT-proBNP levels (Table 5).

**Table 5:** Multivariable Logistic Regression Predicting Elevated NT-proBNP (>3000 pg/mL) Multivariable logistic regression model for predictors of elevated NT-proBNP (>3000 pg/mL). Wald χ² statistics are reported for each predictor. HFrEF: heart failure with reduced ejection fraction; NT-proBNP: N-terminal pro-B-type natriuretic peptide; LVEF: left ventricular ejection fraction; eGFR: estimated glomerular filtration rate

Predictor Variable	Adjusted Odds Ratio (AOR)	95% Confidence Interval	Wald χ²	P-value
Age (per 10 years increase)	1.28	1.05-1.64	5.41	0.02
Male sex	1.42	0.81-2.48	1.59	0.21
Hypertension	1.17	0.66-2.07	0.30	0.58
Diabetes mellitus	1.33	0.74-2.40	0.91	0.34
Ischemic heart disease	2.11	1.18-3.79	6.32	0.01
Atrial fibrillation	2.65	1.22-5.73	6.63	0.01
LVEF <40% (HFrEF)	3.42	1.82-6.46	14.9	<0.001
Renal dysfunction (eGFR <60)	2.88	1.59-5.23	12.7	<0.001

## Discussion

This retrospective study evaluated the association between NT-proBNP levels and LVEF in patients with HF. Out of 165 patients studied, approximately 50% of patients came in with reduced ejection fraction; the rest were distributed between the mildly reduced EF and preserved EF groups. The mean NT-proBNP was much greater in patients with HFrEF than in patients with HFmrEF and HFpEF, and the inverse relationship was strong between NT-proBNP and LVEF in the total cohort. Significantly, subgroup analysis revealed that elevated NT-proBNP levels were also independently linked to atrial fibrillation, renal dysfunction, and ischemic heart disease, and logistic regression indicated that HFrEF and impaired renal function were the strongest predictors of high NT-proBNP levels. We believe that our results support the clinical utility of NT-proBNP as a ventricular dysfunction and global cardiac stress biomarker. The vastly greater NT-proBNP levels in HFrEF patients are consistent with the long-standing pathophysiological principle that dysfunctional systolic performance and chamber expansion elevate wall stress, which provokes the increase of natriuretic peptides [15]. This inverse relationship has been repeatedly found in past research where NT-proBNP levels have been seen to be inversely related to the extent of disease, hospitalization, and mortality in systolic HF patients [16]. However, our results also show the inadequacy of LVEF as a single cardiac dysfunction measure. Even in patients with preserved ejection fraction (HFpEF), NT-proBNP levels were significantly elevated in our study group. This suggests that diastolic dysfunction, increased filling pressures, and systemic comorbidities contribute to the elevation in HFpEF. The analysis of subgroups provides more details regarding the conditions influencing NT-proBNP, but not LVEF [17]. The levels of peptides were also significantly increased in atrial fibrillation, which confirms the previous reports that atrial stretch and atrial contraction loss were the major initiators of natriuretic peptide secretion. Similarly, compromised clearance and crossed hemodynamic load of cardiorenal syndrome were revealed as predictors of elevated NT-proBNP [18]. The findings highlight the importance of a contextual interpretation of the NT-proBNP values, particularly in patients who have comorbidities that may confound its association with ventricular functioning. LVEF is a structural cardiac outcome that measures systolic performance; however, NT-proBNP integrates the load of the hemodynamic strain, neurohormonal trauma, and comorbidity that, combined, may be more efficient in assessing the severity of HF [19].

Clinically, we propose that NT-proBNP would be applicable in conjunction with echocardiographic assessment to optimize diagnosis and risk selection. The high-risk patients who can be provided with the increased guideline-driven therapy and close follow-up are strongly identified by elevated NT-proBNP in the context of lower EF [20,21]. On the other hand, the high NT-proBNP in HFpEF patients underlines the high morbidity of this pattern and the necessity of closer observation even in the face of stable systolic performance. In addition, the addition of comorbid conditions like renal impairment and atrial fibrillation to the analysis of NT-proBNP findings can enhance precision and minimize the error of misclassification [22]. Although this was a single-center study, it involved a relatively large and well-characterized real-world cohort. The patients were stratified according to LVEF categories, allowing meaningful subgroup analysis. Additionally, multivariate confounder adjustment was performed to enhance the robustness and internal validity of the findings.

Limitations

Nevertheless, this study has several limitations. First, as a retrospective single-center analysis, it is subject to selection bias, and residual confounding cannot be fully excluded; thus, the findings may not be entirely generalizable to broader or more diverse populations. Second, NT-proBNP levels were measured at a single time point, limiting evaluation of longitudinal trends or treatment effects. Third, important clinical outcomes such as hospitalization and mortality were not assessed, restricting direct prognostic interpretation. Finally, detailed diastolic function indices and LV strain data were unavailable for all patients, precluding assessment of myocardial relaxation and subclinical systolic dysfunction. Future prospective multicenter studies incorporating these advanced echocardiographic parameters are warranted to provide a more comprehensive understanding of cardiac dysfunction.

## Conclusions

Our findings demonstrate that NT-proBNP levels closely mirror the severity of left ventricular dysfunction, with markedly higher concentrations observed in patients with reduced ejection fraction. Interestingly, elevated NT-proBNP levels were also noted in those with preserved ejection fraction, likely reflecting diastolic dysfunction, increased ventricular stiffness, and elevated filling pressures that contribute to myocardial wall stress even in the absence of overt systolic impairment. This study is unique in that it systematically compared NT-proBNP across all HF phenotypes within a real-world, clinically stratified cohort, while adjusting for major confounders such as age, ischemic heart disease, and atrial fibrillation. By demonstrating a clear gradient of association between NT-proBNP and LVEF categories, our results provide additional evidence supporting its role as a comprehensive marker of overall cardiac burden rather than a purely systolic biomarker.

When interpreted alongside echocardiographic indices, NT-proBNP enhances diagnostic accuracy, refines patient risk stratification, and supports individualized therapeutic decision-making. Future multicenter prospective studies incorporating key clinical outcomes such as mortality and rehospitalization are warranted to validate these findings and explore the prognostic value of NT-proBNP across diverse populations and HF subtypes.

## References

[1] Lazar-Poloczek E, Romuk E, Jacheć W, Wróbel-Nowicka K, Świętek A, Wojciechowska C (2024). Association of NT-proBNP and sST2 with left ventricular ejection fraction and oxidative stress in patients with stable dilated cardiomyopathy. Biomedicines.

[2] Heidenreich PA, Bozkurt B, Aguilar D (2022). 2022 AHA/ACC/HFSA guideline for the management of heart failure: a report of the American College of Cardiology/American Heart Association Joint Committee on clinical practice guidelines. Circulation.

[3] Richards AM (2018). ST2 and prognosis in chronic heart failure. J Am Coll Cardiol.

[4] van Kimmenade RR, Januzzi JL Jr, Ellinor PT (2006). Utility of amino-terminal pro-brain natriuretic peptide, galectin-3, and apelin for the evaluation of patients with acute heart failure. J Am Coll Cardiol.

[5] Ahmad T, Fiuzat M, Neely B (2014). Biomarkers of myocardial stress and fibrosis as predictors of mode of death in patients with chronic heart failure. JACC Heart Fail.

[6] Nymo SH, Aukrust P, Kjekshus J (2017). Limited added value of circulating inflammatory biomarkers in chronic heart failure. JACC Heart Fail.

[7] Badianyama M, Mpanya D, Adamu U, Sigauke F, Nel S, Tsabedze N (2022). New biomarkers and their potential role in heart failure treatment optimisation-an African perspective. J Cardiovasc Dev Dis.

[8] Kakkar R, Hei H, Dobner S, Lee RT (2012). Interleukin 33 as a mechanically responsive cytokine secreted by living cells. J Biol Chem.

[9] Weinberg EO, Shimpo M, De Keulenaer GW (2002). Expression and regulation of ST2, an interleukin-1 receptor family member, in cardiomyocytes and myocardial infarction. Circulation.

[10] Sanada S, Hakuno D, Higgins LJ, Schreiter ER, McKenzie AN, Lee RT (2007). IL-33 and ST2 comprise a critical biomechanically induced and cardioprotective signaling system. J Clin Invest.

[11] Aimo A, Januzzi JL Jr, Bayes-Genis A, Vergaro G, Sciarrone P, Passino C, Emdin M (2019). Clinical and prognostic significance of sST2 in heart failure: JACC review topic of the week. J Am Coll Cardiol.

[12] Januzzi JL, Mebazaa A, Di Somma S (2015). ST2 and prognosis in acutely decompensated heart failure: the International ST2 Consensus Panel. Am J Cardiol.

[13] Schmitz J, Owyang A, Oldham E (2005). IL-33, an interleukin-1-like cytokine that signals via the IL-1 receptor-related protein ST2 and induces T helper type 2-associated cytokines. Immunity.

[14] Chen YY, Borkowski P, Nazarenko N (2024). Demographic and clinical characteristics of New York City Health + Hospitals HIV Heart Failure (NYC4H cohort): cohort profile. BMJ Open.

[15] Shaddy R, Gong J, Garito T (2025). Association between NT‐proBNP changes and clinical outcomes in paediatric patients with heart failure: insights from PANORAMA‐HF and PARADIGM‐HF. ESC Heart Fail.

[16] Tengiz İ, Türk UÖ, Alioğlu E (2013). The relationship between adiponectin, NT-pro-BNP and left ventricular ejection fraction in non-cachectic patients with systolic heart failure: an observational study. Anadolu Kardiyol Derg.

[17] Ziaie N, Ezoji K, Ziaei SG, Chehrazi M, Maleh PA, Pourkia R, Seyfi S (2022). The relationship between N-terminal pro-brain natriuretic peptide (NT-proBNP) levels and diastolic heart failure in patients with COVID-19. Int J Cardiovasc Imaging.

[18] Dhinakaran K, Selvarajan N (2023). Correlation of NT-proBNP levels with clinical and echocardiographic features in evaluation of patients admitted with heart failure. J Med Sci Res.

[19] Arul JC, Raja Beem SS, Parthasarathy M, Kuppusamy MK, Rajamani K, Silambanan S (2025). Association of microRNA-210-3p with NT-proBNP, sST2, and galectin-3 in heart failure patients with preserved and reduced ejection fraction: a cross-sectional study. PLoS One.

[20] Chrysohoou C, Konstantinou K, Tsioufis K (2024). The role of NT-proBNP levels in the diagnosis and treatment of heart failure with preserved ejection fraction-it is not always a hide-and-seek game. J Cardiovasc Dev Dis.

[21] Chen YY, Liang L, Tian PC (2023). Clinical characteristics and outcomes of patients hospitalized with heart failure with preserved ejection fraction and low NT-proBNP levels. Medicine (Baltimore).

[22] Zile MR, Claggett BL, Prescott MF (2016). Prognostic implications of changes in N-terminal pro-B-type natriuretic peptide in patients with heart failure. J Am Coll Cardiol.

